# Introducing transparent peer review to *RSC Advances*

**DOI:** 10.1039/d3ra90060f

**Published:** 2023-07-19

**Authors:** Laura Fisher, Russell J. Cox, Karen Faulds

## Abstract

*RSC Advances* is introducing the option of transparent peer review for authors. Editors-in-Chief Russell J. Cox and Karen Faulds, and Executive Editor Laura C. Fisher offer more detail on how this will work.
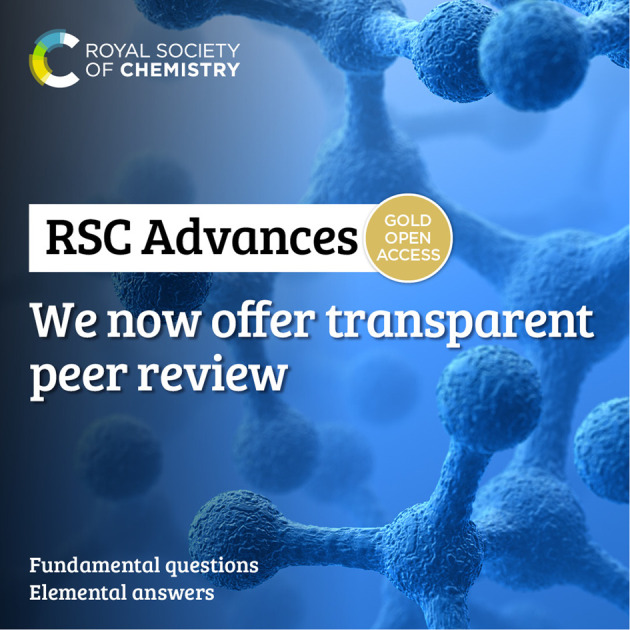

At *RSC Advances* we are committed to ensuring trust and rigour in our peer review processes. In recent years, incidents such as manipulated peer review and the publication of papers believed to be from paper mills^1^ have undermined trust in the peer review process, and we have been working to re-establish this trust. As an open access journal, we also have a commitment to supporting the principles of open science, including greater access to knowledge and data, which helps us promote a more transparent research and publishing culture.^2^

*RSC Advances* is the RSC's largest journal and has always tried to be innovative. In 2017, we were the first RSC journal to convert to an APC-based open access model.^3^ Our article-based publication process gives authors fast and immediate page numbers, our topic-modelling system characterises our papers by subject area so that readers can find what they are looking for in such a broad chemistry journal, and we pioneered the use of Proof Central to simplify the proofreading process.^4^ Our Reviewer Panel enables us to quickly find reliable reviewers, as well as giving the opportunity for early career and under-represented groups to review,^5^ and our recently launched Student Paper Awards recognise students who have made a significant contribution to published work.^6^

It seemed obvious that we should look for the next innovation, something that can help support peer review rigour, and the principles of open science, as well as benefitting our authors and readers. Therefore, we are excited to announce that we are now giving our authors the option of transparent peer review – where anonymous reviewers' comments, editor's decision letters, and the authors' own responses to these are published alongside the main article.^7^

This follows successful trials of transparent peer review in several RSC journals in recent years, as well as its recent introduction to *Chemical Science*.^8^ Not only will this provide readers with an insight into the peer review process for each article, but we hope it will also foster greater trust in the peer review process. Furthermore, we truly believe it will encourage more thorough and constructive reviewer reports, providing authors with more support to improve their articles ahead of publication as well as improving the quality of peer review.

Authors are free to opt in or out of transparent peer review upon submission as well as at any decision stage throughout the process, and reviewers will be informed that their anonymous comments will be published alongside the article should the authors choose to opt-in. While *RSC Advances* does have an article processing charge, there will be no further charge for transparent peer review. More information about transparent peer review, including a FAQs document, can be found on the RSC website,^7^ and examples of transparent peer review in action can be found in *RSC Chemical Biology*, *Digital Discovery*, *Environmental Science: Atmospheres*, *Energy Advances*, *Environmental Science: Advances*, *Sustainable Food Technology*, *EES Catalysis*, *RSC Applied Polymers*, *RSC Applied Interfaces*, and *Chemical Science*.

“We are very excited to introduce the opportunity to take part in transparent peer review to our authors and reviewers. We believe this will bring great benefits in terms of improving the quality of peer review and making the peer review process as transparent as possible for the authors, reviewers, and readers of the journal.” – Karen Faulds, Editor-in-Chief, *RSC Advances*

“Transparent Peer Review shines a light on the reviewing process, while still allowing reviewers to maintain their anonymity. We hope this will allow our readers to have a better insight into the reviewing and revision processes, and encourage reviewers to give better quality and more helpful reviews that will, in turn, help authors. Overall, our aim is to improve transparency and benefit authors, readers and reviewers.” – Russell Cox, Editor-in-Chief, *RSC Advances*

Russell J. Cox, Editor-in-Chief, Leibniz Universität Hannover, Germany

Karen Faulds, Editor-in-Chief, University of Strathclyde, UK

Laura C. Fisher, Executive Editor, *RSC Advances*

## Supplementary Material
